# Supplementary material for: The Association between Nursing Skill Mix and Patient Outcomes in a Mental Health Setting: An Observational Feasibility Study
Source: Int J Environ Res Public Health. 2023 Feb 3;20(3):2715. doi: 10.3390/ijerph20032715 (PMC9915260; doi:10.3390/ijerph20032715)
Supplement: Supplementary file 1 [file ijerph-20-02715-s001.zip › ijerph-2119547-Supplementary Documents S2 and S3.pdf]

Document S2. Patient and nurse data linked

Patient	Age	Sex	Employment status	Primary psychiatric diagnosis	Psychiatric comorbidities	Substance use	Physical comorbidities	Admission date	Admitted under mental health law	On community treatment order prior to admission	Discharge date	Discharge on a community treatment order	Readmitted to inpatient psychiatric services at the participating health service within 12 months of the follow-up period	Readmission date	Readmitted to the inpatient psychiatric ward of another health service	Number of readmissions	HoNos (Health of national outcome Scale) total score on admission to hospital	HoNOS (Health of national outcome scale) total score prior to discharge	Nurse skill mix*
X	27	Male	No	Schizophrenia	Yes	Yes	No	20/01/2020	Yes	No	24/01/2020	Yes	Yes	12/12/2020	No	1	14	6	2

Note *: Mental health nurse to comprehensive nurse ratio for the five days patient X was admitted to the ward

Document S3. Nurses to patient ratio

Staff measure	The total number across the study period (median)	The total number of patients across the study period (median)	Median ratio
Mental health nurse-to-patient	1135 (19)	1913 (32)	1
Comprehensive nurse-to-patient	312 (5)	1913 (32)	0
All nurses-to-patients	1950 (32)	1913 (32)	1
